# Inhibition of chronic lymphocytic leukemia progression by full-length chromogranin A and its N-terminal fragment in mouse models

**DOI:** 10.18632/oncotarget.9407

**Published:** 2016-05-17

**Authors:** Mimma Bianco, Anna Gasparri, Luca Generoso, Emma Assi, Barbara Colombo, Lydia Scarfò, Maria T.S. Bertilaccio, Cristina Scielzo, Pamela Ranghetti, Eleonora Dondossola, Maurilio Ponzoni, Federico Caligaris-Cappio, Paolo Ghia, Angelo Corti

**Affiliations:** ^1^ Tumor Biology and Vascular Targeting Unit, Division of Experimental Oncology, IRCCS San Raffaele Scientific Institute, Milan 20132, Italy; ^2^ B Cell Neoplasia Unit, Division of Experimental Oncology, IRCCS San Raffaele Scientific Institute, Milan 20132, Italy; ^3^ Clinical Lymphoma Unit, Department of Onco-Hematology, San Raffaele Hospital, Milan 20132, Italy; ^4^ San Raffaele Vita-Salute University, Milan 20132, Italy; ^5^ Lymphoid Malignancies Unit, Division of Experimental Oncology, IRCCS San Raffaele Scientific Institute, Milan 20132, Italy

**Keywords:** chromogranin A, vasostatin-1, chronic lymphocytic leukemia, tumor cell trafficking, endothelial barrier function

## Abstract

Chronic lymphocytic leukemia (CLL) is characterized by the accumulation of leukemic B cells in peripheral blood, bone marrow (BM) and lymphoid tissues, and by their recirculation between these compartments. We observed that circulating chromogranin A (CgA) and its N-terminal fragment (called vasostatin-1, CgA_1-76_), two neuroendocrine secretory polypeptides that enhance the endothelial barrier function, are present in variable amounts in the blood of CLL patients. Studies in animal models showed that daily administration of full-length human CgA_1-439_ (0.3 μg, i.v., or 1.5 μg/mouse, i.p.) can reduce the BM/blood ratio of leukemic cells in Eμ-TCL1 mice, a transgenic model, and decrease BM, lung and kidney infiltration in Rag2^−/−^γc^−/−^ mice engrafted with human MEC1 CLL cells, a xenograft model. This treatment also reduced the loss of body weight and improved animal motility. *In vitro*, CgA enhanced the endothelial barrier integrity and the trans-endothelial migration of MEC1 cells, with a bimodal dose-response curve. Vasostatin-1, but not a larger fragment consisting of N-terminal and central regions of CgA (CgA_1-373_), inhibited CLL progression in the xenograft model, suggesting that the C-terminal region is crucial for CgA activity and that the N-terminal domain contains a site that is activated by proteolytic cleavage. These findings suggest that circulating full-length CgA and its fragments may contribute to regulate leukemic cell trafficking and reduce tissue infiltration in CLL.

## INTRODUCTION

Human chromogranin A (CgA) is a 439-residue long protein stored in the secretory granules of many (neuro)endocrine cells and neurons and released in the blood of normal subjects at sub-nanomolar levels [1, 2]. Increased circulating levels have been detected in patients with neuroendocrine tumors, heart failure, renal failure, hypertension, atrophic gastritis, rheumatoid arthritis, arteritis and other inflammatory conditions [2–6], or in subjects treated with proton pump inhibitors (PPI), a class of drugs commonly used to treat acid peptic disorders [7–11].

CgA has an intracellular function in secretory granule biogenesis [12, 13], as well as important extracellular functions as a precursor of various bioactive peptides involved in the regulation of metabolism, innate immunity, cardiovascular system, vascular contractility, and endothelial barrier function [1, 2]. A growing body of evidence suggests that CgA and its N-terminal fragment 1-76, called vasostatin-1 (VS-1), can also have a role in tumor biology. For example, we have recently shown that circulating CgA can regulate angiogenesis and tumor growth in various models of solid tumors [14–16]. Structure-function studies have shown that full-length CgA contains a) a latent anti-angiogenic site in the N-terminal region 1-76, b) a latent pro-angiogenic site in the region 352-373 and c) a functional anti-angiogenic site in the C-terminal region 410-439 [14, 16, 17]. The latent anti-angiogenic site is activated by cleavage of the Q_76_-K_77_ peptide bond, by unknown enzymes, whereas the pro-angiogenic site can be activated by thrombin- or plasmin-mediated cleavage of the R_373_-R_374_ dibasic site [14, 18]. Furthermore, using murine models of mouse mammary adenocarcinomas and melanomas we have shown that full-length CgA and VS-1 can enhance the endothelial barrier function and reduce the trans-endothelial migration of tumor cells, thereby reducing the trafficking of cancer cells from tumor-to-blood and from blood-to-tumor/normal tissues (i.e. the metastasis and the tumor “self-seeding” processes) [19, 20].

These notions prompted us to investigate the role of circulating CgA in chronic lymphocytic leukemia (CLL), a hematological cancer characterized by the progressive accumulation of CD5^+^ leukemia B cells in peripheral blood, bone marrow and lymphoid tissues, and by the continuous trafficking of these cells between these tissue compartments [21]. To this aim we have analyzed the levels of CgA and VS-1 in the plasma of CLL patients, and investigated their effects on CLL progression in Eμ-TCL1 mice, a transgenic model of CLL [22] and in Rag2^−/−^γc^−/−^ mice engrafted with human MEC1 CLL cells, a xenograft model [23]. We show that circulating CgA and its N-terminal fragment are present in variable amounts in the blood of CLL patients. Furthermore, using the mouse models, we show that physiologically relevant concentrations of full-length CgA and VS-1, but not of fragments lacking the C-terminal region, can reduce the trafficking of CLL cells in blood and tissues and delay CLL progression. Finally, we provide experimental evidence suggesting that an important mechanism of action of full-length CgA is based on the regulation of CLL cell migration through the endothelial barrier.

## RESULTS

### CgA plasma levels are increased in CLL patients

**Figure 1:**
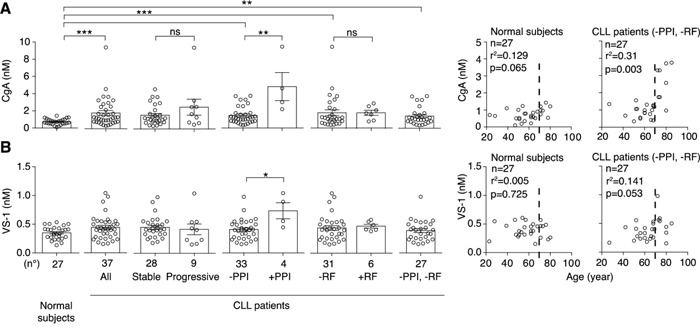
Levels of CgA and VS-1 in plasma samples obtained from normal subjects and CLL patients Left panels: plasma levels of CgA **A.** and VS-1 **B.** in 27 normal subjects and 37 CLL patients with stable (n=28) or progressive (n=9) disease, as measured by ELISA. Plasma levels in patients treated or not with proton pump inhibitors (PPI) and with or without renal failure (RF) are also shown. Bars (mean ± SEM). *, P < 0.05; **, P < 0.01; ***, P < 0.001 (Mann-Whitney test). Right panels: Spearman correlation between age and CgA or VS-1 in patients untreated with PPI and without RF (n= 27).

We measured CgA in plasma samples from 37 patients at diagnosis and 27 normal subjects, by ELISA. The results show that CLL patients had increased plasma levels of CgA, compared to normal subjects, particularly those >70-year-old or those treated with proton pump inhibitors (Figure 1A). Of note, one patient treated with PPI (age 64 years) had a CgA plasma level about 20-fold higher than the average normal value. Thus, a wide range of concentrations of CgA (0.36-9.45 nM), including abnormal values, was observed in this study population. No significant association with biological parameters (CD38, ZAP70, IGHV gene mutational status) or with disease progression was observed.

When we excluded patients treated with PPI or with renal failure (RF), two conditions known to be associated with increased CgA levels [2, 4–11, 27], we still observed a significant increase of CgA (Figure 1A), suggesting that CLL is a condition *per se* sufficient to enhance the CgA levels.

To assess whether leukemic cells could secrete CgA, we purified CD5^+^ CD19^+^ cells from the peripheral blood of five patients having high plasma levels of CgA, cultured them for 6 days *in vitro*, and measured the CgA levels in the supernatant (collected every day) by ELISA. No CgA was detected at any time, arguing against the hypothesis that leukemic cells were the source of abnormal plasma CgA. More likely, CgA was released in the circulation by the neuroendocrine system.

### VS-1 plasma levels are increased in CLL patients treated with PPI, but not in untreated patients

The levels of VS-1 in the blood of normal subjects and CLL patients were analysed using a very selective assay, unable to cross-react with full-length CgA nor with larger fragments [14]. No significant difference of VS-1 was observed between controls and patients, except in those treated with PPI (Figure 1B). Thus, the release of VS-1 did not mirror the release of CgA in patients not treated with PPI.

### CgA plasma levels are increased in Eμ-TCL1 mice, a transgenic model of CLL

**Figure 2:**
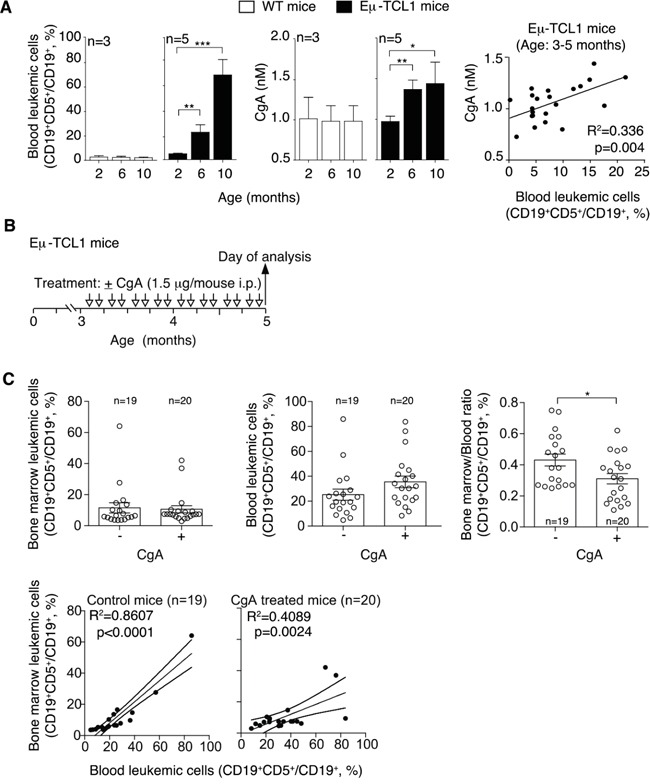
Plasma levels of CgA in Eμ-TCL1 mice and effect of exogenous CgA on the distribution leukemic cells in different compartments **A.** Left panels: percentage of leukemic cells (CD19^+^ CD5^+^) in the circulating B-cell population (CD19^+^) of Eμ-TCL1 transgenic mice and non-transgenic littermates at different ages (two, six and ten months), as determined by FACS analysis. Central panels: CgA plasma levels, as measured by ELISA, in the same mice. Right panel: linear regression between blood leukemic cells and CgA in 3-5 month-old Eμ-TCL1 mice. **B.** Schematic representation of the experiment. Three-month-old Eμ-TCL1 mice were injected with 1.5 μg of CgA or with vehicle alone (i.p., bi-weekly, for 2 month). **C.** Upper panels: leukemic cell population in the bone marrow and blood of Eμ-TCL1 mice treated with vehicle (−) or with CgA (+). The bone marrow/blood ratio of leukemic cells is also shown. Bottom panels: linear regression (with 95% confidence interval) between peripheral blood and bone marrow leukemic cells. (A, C) Bars (mean ± SEM); *, p<0.05; **, p<0.01; ***, P < 0.001 by two tailed *t* test.

To assess whether CLL is indeed a condition *per se* sufficient to enhance the plasma levels of this protein we monitored the circulating levels of CgA in Eμ-TCL1 mice, a transgenic mouse model of CLL [23]. Using an assay specific for murine CgA we observed a progressive increase of circulating CgA in these mice, but not in age-matched control mice (Figure 2A). Interestingly, CgA significantly correlated with the concentration of leukemic cells in the blood of 3-5 month-old mice (Figure 2A, right panel). As these mice were not treated with drugs, these findings suggest that the presence of CLL is a condition *per se* sufficient to enhance the CgA levels.

### CgA reduces the bone marrow/blood ratio of leukemic cells in Eμ-TCL1 mice

To assess whether circulating CgA might influence the behavior of CLL cells we studied the effect of CgA on the distribution of leukemic cells in the blood and the bone marrow (BM) of Eμ-TCL1 transgenic mice. To this aim, 3-month-old mice (i.e. mice with CgA values in the normal range) were treated bi-weekly with intra-peritoneal injections of 1.5 μg of full-length CgA or saline solution only (Figure 2B). This dose, when given i.p., generates peak plasma levels of about 3-4 nM CgA that progressively declines to 0.5-1 nM in 7-8 h, as measured by ELISA, i.e. levels similar to those found in CLL patients. After two months, we sacrificed the mice and measured the percentage of leukemic cells in blood and BM, by FACS analysis with anti-CD5 and anti-CD19 antibodies. Although no significant changes of the percentage of CD19^+^CD5^+^ (leukemic cells) over the total CD19^+^ cells (B-cells) were observed in the BM and in the blood of treated mice versus controls, a significant reduction of the BM/blood ratio of CLL cells was apparent (Figure 2C). Similarly, while in untreated mice the leukemic cells in the blood strongly correlated with leukemic cells in the BM (r^2^=0.86; p<0.0001; regression line slope=0.68 ± 0.07), a weaker correlation and a lower slope of the regression line was observed in CgA-treated mice (r^2^=0.41; p<0.01; slope= 0.32 ± 0.09). Thus, the blood leukemic cells were associated with less than a half of BM leukemic cells in CgA-treated compared to untreated mice. These findings suggest that full-length CgA may affect the distribution of leukemic cells in these compartments, possibly by affecting cell intra-/extra-vasation and/or by causing differential cell proliferation in these compartments.

### CgA inhibits CLL progression in a xenograft mouse model with a biphasic dose-response curve

**Figure 3:**
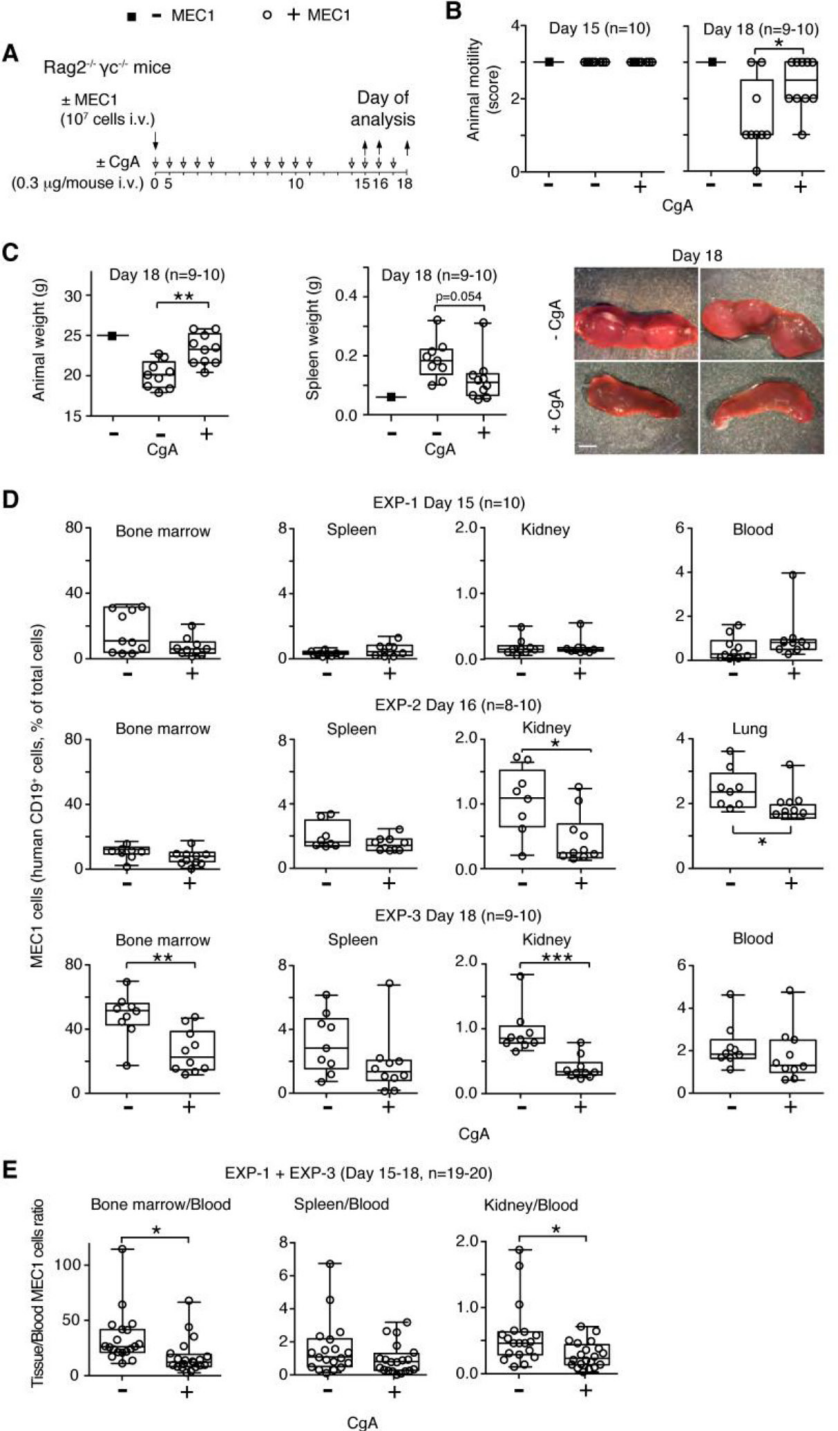
Effect of low-dose CgA on disease progression and organ infiltration by leukemic cells in the MEC1 xenograft model **A.** Schematic representation of the experiment. Rag2^−/−^γc^−/−^ mice were injected, i.v., without (■) or with (○) 10^7^ MEC1 and sacrificed at day 15, 16 or 18, in three independent experiments (EXP-1, -2 and -3). Mice were treated, i.v., with 0.3 μg of CgA (+) or with vehicle (−) at the indicated time (8-10 mice/group). **B.** Animal motility at day 15 and 18, before killing: score 0, impaired motility with a severe inability to reach food and water; score 1, inability to remain upright with hunched abnormal posture; score 2, less vital and curious behavior; score 3, animals showing a normal behavior. **C.** Animal and spleen weights at the indicated time. Photographs of representative spleens at day 18 of mice treated with or without CgA are also shown. **D.** Quantification of MEC1 cells (CD19^+^) in bone marrow, spleen, kidney, lung, and blood at day 15, 16 and 18 (three separate experiments). Cell suspension obtained from different organs were stained with phycoerytrin-cyanine 7-labelled anti-human CD19 antibody and analysed by FACS. **E.** Tissue/blood ratio of MEC1 cells in different organs is shown. (B-E) Box-plots with median, interquartile and 5-95 percentile values; *, P < 0.05; **, P < 0.01; ***, P < 0.001 (*t* test, two-tailed).

To dissect its mechanisms of action and to further assess the role of CgA on CLL cell behavior we then studied the effect of CgA in the MEC1 xenograft model, which is based on the intravenous injection of human MEC1 CLL cells (stably transfected to express GFP) into Rag2^−/−^γc^−/−^ mice [23], thus bypassing the intravasation process. Mice were treated daily from day 0 to 15 with 0.3 μg of human full-length CgA (i.v.) or saline solution only. This dose, when given i.v., generates circulating levels similar to those found in CLL patients (about 3-4 nM; half life, 1 h). Three different experiments were performed, ending at day 15, 16 and 18 (Figure 3A). Disease progression was clearly evident at day 18 as indicated by altered animal motility, loss of body weight, increase of spleen weight, and increase of MEC1 cell infiltration of BM, spleen, lung, and kidney (Figure 3B-3D and Supplemental Figure 1). CgA treatment improved animal motility, reduced the changes of body and spleen weights, and decreased BM, lung and kidney infiltration by MEC1 cells (Figure 3B-3D). These findings suggest that an increase of circulating full-length CgA above the normal levels can delay disease progression. Of note, the BM/blood and kidney/blood ratio were lower in mice treated with CgA compared to controls (Figure 3E).

Interestingly, when we treated mice with a 30-fold higher dose of full-length CgA (10 μg/day) no significant effects were observed (Supplemental Figure 2A-2E), suggesting that CgA can affect CLL progression with a biphasic dose-response curve.

### CgA does not affect BM microvessel density, MEC1 cell proliferation and viability

**Figure 4:**
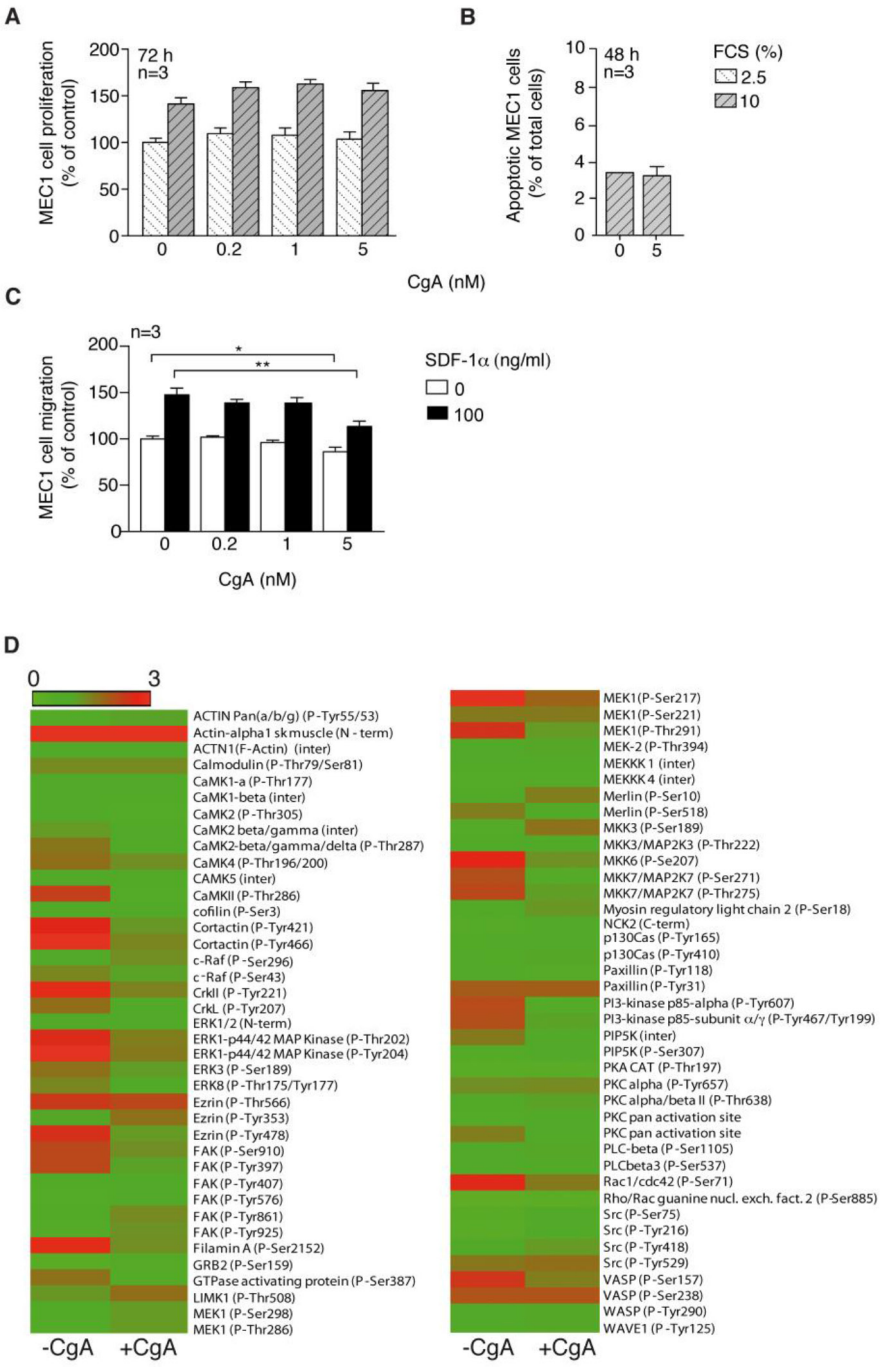
Effect of CgA on MEC1 cell proliferation, viability, migration and cytoskeleton activation **A.** Effect of different concentrations of CgA and FCS on MEC1 cell proliferation (72 h incubation). Bars, mean ± SEM. **B.** Effect of different amounts of CgA on MEC1 cell viability (48 h incubation). Bars, mean ± SEM. **C.** Effect of CgA on the migration of MEC1 cells through trans-well system microporous filters. The upper and lower chambers of trans-well systems, pre-coated with HUVECs, were filled with different amounts of CgA and 0 or 100 ng/ml SDF-1α, respectively. Cells migrated to the lower side of the filter after 4 h of incubation, were counted by FACS (see *Supplemental Methods*). Bars, mean ± SEM; *, p<0.05; **, p<0.01 by two tailed *t* test. **D.** Effect of CgA (5 nM) on protein phosphorylation in MEC1 cells. After treatment, cells were lysed and analyzed with cytoskeleton II Phospho Array (full Moon Byosystems). Visualization of the microarray data was obtained using matrix2png (1.2.1) program (Pavlidis, 2003).

The mechanism of action of the anti-tumor activity of CgA was further investigated. No effect of CgA was observed on MEC1 cell proliferation and viability at 0.2-5 nM concentrations in *in vitro* studies (Figure 4A and 4B). Furthermore, no significant difference was observed in the BM microvessel density of mice treated with low-dose CgA or with vehicle (Supplemental Figure 3). These data argue against the hypothesis that inhibition of cell proliferation (direct or consequent to angiogenesis inhibition) was a primary mechanism of action.

### CgA weakly inhibits MEC1 cell migration *in vitro*

The effect of CgA on MEC1 cell migration was then investigated. Five nanomolar CgA, but not lower concentration, exerted a modest inhibitory effect on MEC1 cell migration through microporous filters, in the presence or the absence of SDF-1α, a chemotactic factor for lymphocytes (Figure 4C). Using the Cytoskeleton Phospho Antibody Array (Full Moon BioSystems) we observed that 5 nM CgA could affect the phosphorylation of various proteins involved in cytoskeleton organization, most in a negative manner (Figure 4D). These data suggest that CgA at a high concentration can affect MEC1 cell biology and weakly inhibit their migration.

### CgA inhibits MEC1 trans-endothelial migration with a U-shaped dose-response curve

**Figure 5:**
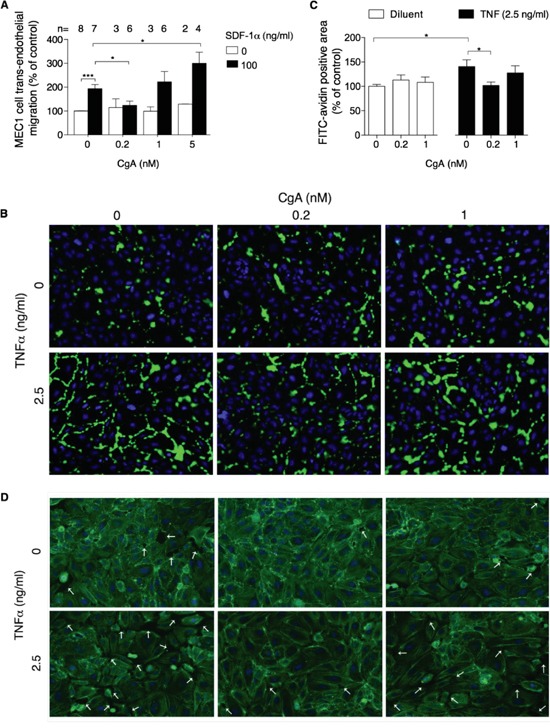
Effect of CgA on MEC1 cell trans-endothelial migration and on endothelial cell permeability **A.** Effect of CgA on the migration of MEC1 cells through endothelial monolayers cultured in trans-well systems. The upper and lower chambers of trans-well systems, pre-coated with HUVECs, were filled with different amounts of CgA and 0 or 100 ng/ml SDF-1α, respectively. Cells migrated to the lower side of the filter after 4 h of incubation, were counted by FACS (see *Supplemental Methods*). Bars, mean ± SEM of the indicated number of independent experiments (n) each in quadruplicate (*, P < 0.05; ***, P < 0.001 by two tailed *t* test). **B.** Effect of CgA on TNFα-induced endothelial cell permeability. The assay was performed as described in *Supplemental Methods*. The binding of FITC-avidin to coverslips coated with biotinylated gelatin and with confluent HUVEC cell monolayers (pre-treated for 4 h with the indicated doses of CgA and TNFα) is shown. Nuclei were stained with 4′,6-diamidino-2-phenylindole (DAPI, blue). Images were taken using 20× objectives. Green fluorescence corresponds to the leaky areas of the endothelial monolayers. **C.** Quantification of FITC-avidin flux through the endothelial monolayers after treatment with CgA and TNFα (see *Supplemental Methods*). Three independent experiments were performed (2 slides/condition, 5 fields/slide for each experiment). Bars, mean ± SEM (*, p<0.05 by two tailed *t* test). **D.** Effect of CgA on F-actin assembly and gap formation (arrows) induced by TNFα in confluent HUVEC. F-actin fibers were stained with FITC-phalloidin (green); nuclei were stained with DAPI (blue). Images were taken using a 20× objective.

We then studied the effect of CgA on MEC1 cell trans-endothelial migration, using microporous filters coated with endothelial cells, in the presence or absence of SDF-1α. In this co-culture experiment, CgA significantly affected the migration of MEC1 cells with a bimodal dose-response curve, with inhibitory effects at 0.2 nM and stimulatory effects at 5 nM (Figure 5A). Considering that 5 nM CgA exerted a modest inhibitory effect on MEC1 cell migration in the absence of endothelial cells and no effects at all at 0.2-1 nM (Figure 4C), it is very likely that the bimodal effect observed in the co-culture experiment were related to a selective effect of CgA on the endothelial barrier. No significant effects on SDF-1α induced migration were observed with VS-1 and CgA_1-373_ (Supplemental Figure 5)

Using a multi-analyte profiling approach on MEC1/endothelial cell co-culture supernatants we observed the presence of various cytokines capable of disrupting the endothelial barrier integrity, such as TNFα, TNFβ, and VEGF (Supplemental Table 2). To assess whether low concentrations of CgA could inhibit the trans-endothelial migration of MEC1 cells by protecting the endothelial barrier integrity we analysed the effect of 0, 0.2 and 1 nM CgA on gap formation induced by TNFα in endothelial cell monolayers. CgA at 0.2 nM concentration, but not 1 nM, inhibited the TNFα-induced F-actin reorganization, gap formation and permeability of endothelial cell monolayers (Figure 5B-5D). These findings suggest that CgA can enhance the endothelial barrier function in a bimodal manner, which may explain its bimodal effect on MEC1 cell trans-endothelial migration.

No enhancement of TNFα, TNFβ, and VEGF production by MEC1/endothelial cell co-culture was observed in the presence of 5 nM CgA (Supplemental Table 2). Thus, the loss of protective activity on endothelial barrier at high dose was not related to enhanced production of these cytokines.

### The C-terminal region of CgA is crucial for its anti-tumor activity

We then tested the fragment CgA_1-373_ in the MEC1 model. Interestingly, no significant effects were observed with daily administration (i.v.) of 0.24 μg of CgA_1-373_ (equivalent to the bioactive dose of full-length CgA and VS-1 on a molar basis) (Supplemental Figure 4A-4D), suggesting that the anti-leukemic activity of full-length CgA requires an intact C-terminal region (374-349). Possibly, this region contains the bioactive site or is crucial for the function of a site located elsewhere.

### The N-terminal fragment of CgA (VS-1) inhibits CLL progression in the CLL xenograft model

**Figure 6:**
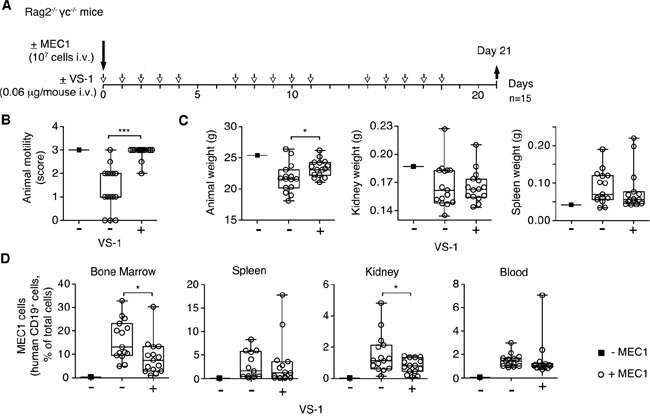
Effect of low-dose VS-1 on organ infiltration by leukemic cells and disease progression in the MEC1 xenograft model **A.** Schematic representation of the experiment. Rag2^−/−^γc^−/−^ mice, injected without (■) or with (○) MEC1 cells (i.v.), were treated with vehicle (−) or with 0.06 μg of VS-1 (+) at the indicated time, and sacrificed at day 21. **B.** Animal motility at day 21 before killing (see the legend of Figure 3 for score explanation). **C.** Animal weight, kidney and spleen weights at day 21. **D.** Quantification of MEC1 cells in bone marrow, spleen, kidney, lung, and blood at day 21. (B-D) Box-plots with median, interquartile and 5-95 percentile values; *, P < 0.05; ***, P < 0.001 (*t* test, two-tailed).

Finally, we investigated the effects of VS-1 in the MEC1 model. Daily administration (i.v.) of 0.06 μg of VS-1 (i.e. a dose equivalent, on a molar basis, to the bioactive dose of full-length CgA) exerted anti-tumor effects similar to those induced by full-length CgA (Figure 6A-6D). These data suggest that also this fragment, which is present in variable amounts in the blood of CLL patients, can contribute to delay CLL progression. Furthermore, considering that CgA_1-373_ includes the CgA_1-76_ region (corresponding to VS-1) and that an equimolar dose of CgA_1-373_ is inactive in this model these results imply that a latent bioactive site is present in the N-terminal domain, which is activated only after cleavage and liberation of VS-1. Thus, changes in N- and C-terminal processing of CgA may regulate its anti-leukemic activity.

## DISCUSSION

The results of the present study show that a wide range of CgA levels is present in the blood of CLL patients (ranging from 0.36 to 9.45 nM in our study population). Increased CgA levels were likely the consequence of various factors, including CLL, renal failure, and treatment with proton pump inhibitors (PPI), a class of drugs commonly used to treat acid peptic disorders. The effect of PPI was somehow expected, as these drugs are known to cause enterochromaffin-like cell hyperplasia and, consequently, to enhance 2-3-fold, and in certain cases up to 10-30-fold, the circulating levels of CgA [7–11]. Interestingly, when we excluded patients taking PPI and/or having renal failure (another condition known to be associated with increased levels of CgA) we still observed a significant increase of plasma CgA, particularly in >70-year-old patients, but not (or much less) in age-matched control subjects. Furthermore, in Eμ-TCL1 mice, a transgenic model of CLL, we observed a progressive increase of circulating CgA (but not in age-matched control mice) that significantly correlated with the increase of circulating leukemic cells. These findings suggest that CLL is a condition *di per se* sufficient to enhance the CgA levels.

The lack of detectable CgA in the supernatant of leukemic cells isolated from patients argues against the hypothesis that CLL cells were a primary source of the their abnormal circulating CgA. More likely, CgA was released in circulation by the neuroendocrine system, possibly as a consequence of stress-induced neuroendocrine activation.

The question arises as to whether different levels of circulating CgA in CLL patients might influence disease progression. The results of our *in vivo* studies performed in the murine Eμ-TCL1 transgenic model and in the MEC1 xenograft model support this hypothesis. Indeed, bi-weekly injection of physiologically relevant doses of recombinant full-length CgA to Eμ-TCL1 mice significantly reduced the BM/blood ratio of leukemic cells. Furthermore, daily treatment of the Rag2^−/−^γc^−/−^ engrafted with MEC1 cells improved animal motility, reduced the loss of body weight, and decreased bone marrow, lung and kidney infiltration by leukemic cells. These observations suggest that changes in the circulating levels of full-length CgA can regulate disease progression.

The results of mechanistic studies show that full-length CgA barely inhibits, or not at all, *in vitro* MEC1 cell proliferation, viability and migration through microporous filters, whereas it can inhibit cell migration through endothelial cell monolayers. Interestingly, in trans-endothelial migration assays CgA exerted inhibitory effects when added at 0.2 nM concentration, and stimulatory effects when added at 5 nM, pointing to bimodal mechanisms. The bimodal regulation of the trans-endothelial migration of MEC1 cells may explain the antitumor effects of low-dose CgA (0.3 μg/day) and the lack of effects observed with high-dose CgA (10 μg/day) *in vivo*. According to this hypothesis, in the MEC1 model we observed that mice treated with low-dose CgA (0.3 μg/day), but not with the high-dose, had a lower BM/blood and kidney/blood ratios of MEC1 cells compared to vehicle-treated mice.

In previous studies we have shown that low concentrations of CgA can reduce the trans-endothelial migration of other malignant cells, such as TSA mammary adenocarcinoma and B16 melanoma cells, by enhancing endothelial VE-cadherin dependent adherence junctions [20, 28]. In these studies low-dose CgA could significantly reduce the formation of TSA and B16 cell colonies in the lungs of mice injected with these cells in the tail vein [20]. Furthermore, in the TSA model a two-fold increase of endogenous circulating CgA caused by treatment with PPI was sufficient to reduce lung colony formation, an effect that was inhibited by anti-CgA antibodies [20]. Based on these findings and on the results of the present study it is tempting to speculate that the presence of circulating full-length CgA at concentrations close or slightly above the physiological values is beneficial for patients with CLL, as it may reduce the trans-endothelial trafficking of neoplastic cells from blood to tissues and *vice versa*. However, considering the bimodal effects of CgA, we cannot exclude that a marked increase of circulating CgA, as observed in certain patients, may actually be detrimental.

The results of structure-activity studies performed with full-length CgA and CgA_1-373_ fragment suggest that functional CgA requires an intact C-terminal region, as a dose of CgA_1-373_ equivalent to the bioactive dose of full-length CgA (on a molar basis) was inactive in the MEC1 model. Furthermore, the lack of activity observed with CgA_1-373_ and the positive activity observed with the N-terminal fragment CgA_1-76_ (VS-1) suggest that proteolytic cleavage at residue Q_76_ can activate a functional site located in the N-terminal domain. Remarkably, we observed that this fragment is present in the blood of CLL patients in variable amounts (0.153 to 1.04 nM). Considering that VS-1 can enhance the endothelial barrier function and inhibit angiogenesis in a variety of *in vitro* and *in vivo* assays in the 0.2-5 nM range [14, 16] it is possible that this fragment contributes, together with full-length CgA, to reduce CLL cell trafficking and organ infiltration in patients. The proteases responsible for CgA cleavage in patients are unknown.

In conclusion, the results of the present study suggest that circulating CgA may reduce leukemic cell trafficking and disease progression in CLL patients depending on its concentration and proteolytic processing. Monitoring CgA and CgA-derived polypeptides in patients before and after therapy with PPI or other drugs, and the evaluation of their prognostic value, may provide important information for the management of CLL. Finally, the anti-tumor activity observed with exogenous CgA and VS-1 in the animal models suggest that pharmacological enhancement of circulating CgA and/or VS-1 in patients might represent potential therapeutic strategies that merit further investigation.

## MATERIALS AND METHODS

### Patients and plasma samples

Peripheral blood plasma samples were obtained from 37 patients with CLL at diagnosis after informed written consent and with full ethical approval from the institutional review board (males/females, 23/14; stable/progressive disease, 28/9; age, 66.2 ± 12.2 years (mean ± SD)). Demographic and clinical data regarding the study population are reported in Supplemental Methods and Supplemental Table 1.

### Cell lines

MEC1 cells were obtained from Deutsche Sammlung von Mikroorganismen und Zellkulturen (DMSZ, Germany) and cultured in RPMI 1640 medium (Lonza) supplemented with 10% fetal bovine serum, 2 mM glutamine and 15 μg/ml gentamicin (Sigma Aldrich). MEC1 cells stably expressing green fluorescent protein were generated as described previously [23] and cultured in supplemented RPMI 1640 medium, as above, containing 5 μg/ml blasticidin (Life Technologies). Human umbilical vein endothelial cells (HUVECs; Lonza, Switzerland; passage 5 to 8) were cultured in EGM™-2 BulletKit™ (EBM-2 basal culture medium with 2% FBS plus SingleQuots™ of growth supplements (Lonza)).

Human leukemic lymphocytes were isolated from the peripheral blood of CLL patients, using the human B-cell enrichment cocktail (RosetteSep, StemCell Technologies) or cell separation columns (Miltenyi Biotec). CD19 and CD5 co-expression on cell surface was checked by flow cytometry (FC500, Beckman Coulter) and the purity was always >97%.

### CgA, CgA fragments and antibodies

Full-length human (CgA_1-439_) and murine CgA (CgA_1-445_), human CgA_1-373_ (a fragment lacking the C-terminal region) and human CgA_1-76_ (the N-terminal fragment corresponding to VS-1) were prepared by recombinant DNA technology as described previously [14].

Murine monoclonal antibodies (mAb) B4E11 and 5A8 against CgA (epitopes located within residues 68-71 and 54-57 of human CgA, respectively) were described previously [24, 25]. Both antibodies can efficiently recognize full-length human CgA as well as fragments containing the N-terminal region 1-76. Anti-VS-1 polyclonal antiserum (called α-76) was raised in rabbits by immunization with human CgA_71-76_ peptide coupled to keyhole limpet hemocyanin. This antibody recognizes the fragment human CgA_1-76_ (VS-1) but not larger fragments or full-length CgA [14]. Anti-CgA polyclonal antiserum (called α-FRs) was raised in rabbits by immunization with recombinant human CgA_1-439_. This antibody (immunodominant epitopes 90-133, 163-187, 222-256, 315-338) recognizes full-length human CgA and fragments containing central regions but not CgA_1-76_ (VS-1). A similar antiserum was also produced against murine recombinant CgA.

### CgA and VS-1 immunoassays

Human CgA in plasma samples of patients and normal subjects was detected using a sandwich ELISA based on the use of mAb B4E11 in the capture step, the polyclonal antibody α-FRs in the detection step, and human CgA_1-439_ as a standard, as described previously [5, 15]. This ELISA can detect full-length CgA_1-439_ and fragments containing the N-terminal and part or the entire central and C-terminal regions, but not VS-1.

Murine CgA in plasma samples of mice was measured with a similar sandwich ELISA based on the use of mAb 5A8 in the capture step, the polyclonal anti-murine CgA antibody in the detection step, and recombinant murine CgA_1-445_ as a standard.

VS-1 was detected using a sandwich ELISA based on the use of mAb 5A8 in the capture step, the polyclonal antibody α-76 in the detection step, and recombinant human CgA_1-76_ as a standard, as described previously [15, 26]. This ELISA can detect VS-1 but not larger fragments or full-length CgA_1-439_ [14].

### *In vivo* studies with Eμ-TCL1 transgenic mice

Eμ-TCL1 transgenic mice [22] were kindly provided by Dr. J. Byrd (Ohio State University, Columbus, OH). Three-month-old mice were treated with 1.5 μg of CgA or with vehicle alone (i.p., bi-weekly, for 2 month) and analysed for the presence of leukemic cells in blood and tissues as described in Supplemental Methods.

### *In vivo* studies with Rag2^−/−^γc^−/−^ mice xenografted with MEC1 cells

Rag2^−/−^γc^−/−^ mice were kindly provided by CIEA and Taconic (Japan). Rag2^−/−^γc^−/−^ female mice (8-week-old) were challenged intravenously with 10^7^ MEC1 cells, stably transfected with the green fluorescence protein, in 0.1 ml of physiological solution. Mice were treated daily (i.v.) with equimolar doses of CgA (0.3 μg), VS-1 (0.06 μg) or CgA_1-373_ (0.24 μg) in saline containing 100 μg/ml of human serum albumin, or with vehicle alone. Other mice were treated with a 30-fold higher dose of CgA (10 μg) in the same diluent. Mice were sacrificed at day 15, 16, 18 or 21, and dissected. The presence of leukemic cells in blood and tissues were then analyzed as described in Supplemental Methods.

### Cell proliferation, apoptosis and migration assays

MEC1 cell proliferation, apoptosis, migration and trans-endothelial migration assays were performed as reported in Supplemental Methods.

### *In vitro* vascular permeability imaging assay

The effect of CgA on endothelial permeability was assessed using the “*In vitro vascular permeability imaging assay*” kit (EDM Millipore) as described in Supplemental Methods.

### Statistical analysis

Data are described as bars with mean ± SEM or box-plots with median, interquartile and 5-95 percentile values, as indicated in each figure legend. The Mann-Whitney test was used to assess statistically significant differences between groups of patients. The Spearman test was performed to investigate the association between age and CgA or VS-1 levels. The Student *t* test (two-tailed) was used to evaluate differences between experimental groups of *in vivo* and *in vitro* studies, as indicated in the figure legends.

## SUPPLEMENTARY MATERIALS FIGURES AND TABLES





## References

[1] Helle KB, Corti A, Metz-Boutigue MH, Tota B (2007). The endocrine role for chromogranin A: a prohormone for peptides with regulatory properties. Cell Mol Life Sci.

[2] Loh YP, Cheng Y, Mahata SK, Corti A, Tota B (2012). Chromogranin A and derived peptides in health and disease. J Mol Neurosci.

[3] Zhang D, Lavaux T, Voegeli AC, Lavigne T, Castelain V, Meyer N, Sapin R, Aunis D, Metz-Boutigue MH, Schneider F (2008). Prognostic value of chromogranin A at admission in critically ill patients: a cohort study in a medical intensive care unit. Clin Chem.

[4] Corti A (2010). Chromogranin A and the tumor microenvironment. Cell Mol Neurobiol.

[5] Ceconi C, Ferrari R, Bachetti T, Opasich C, Volterrani M, Colombo B, Parrinello G, Corti A (2002). Chromogranin A in heart failure; a novel neurohumoral factor and a predictor for mortality. Eur Heart J.

[6] Portela-Gomes GM, Grimelius L, Wilander E, Stridsberg M (2010). Granins and granin-related peptides in neuroendocrine tumours. Regul Pept.

[7] Giusti M, Sidoti M, Augeri C, Rabitti C, Minuto F (2004). Effect of short-term treatment with low dosages of the proton-pump inhibitor omeprazole on serum chromogranin A levels in man. Eur J Endocrinol.

[8] Sanduleanu S, Stridsberg M, Jonkers D, Hameeteman W, Biemond I, Lundqvist G, Lamers C, Stockbrugger RW (1999). Serum gastrin and chromogranin A during medium- and long-term acid suppressive therapy: a case-control study. Aliment Pharmacol Ther.

[9] Sanduleanu S, De Bruine A, Stridsberg M, Jonkers D, Biemond I, Hameeteman W, Lundqvist G, Stockbrugger RW (2001). Serum chromogranin A as a screening test for gastric enterochromaffin-like cell hyperplasia during acid-suppressive therapy. Eur J Clin Invest.

[10] Vlasveld LT, van 't Wout J, Castel A (2011). False elevation of chromogranin A due to proton pump inhibitors. Netherl J Med.

[11] Mosli HH, Dennis A, Kocha W, Asher LJ, Van Uum SH (2012). Effect of short-term proton pump inhibitor treatment and its discontinuation on chromogranin A in healthy subjects. J Clin Endocrinol Metab.

[12] Pasqua T, Mahata S, Bandyopadhyay GK, Biswas A, Perkins GA, Sinha-Hikim AP, Goldstein DS, Eiden LE, Mahata SK (2016). Impact of Chromogranin A deficiency on catecholamine storage, catecholamine granule morphology and chromaffin cell energy metabolism *in vivo*. Cell Tissue Res.

[13] Taupenot L, Harper KL, Mahapatra NR, Parmer RJ, Mahata SK, O'Connor DT (2002). Identification of a novel sorting determinant for the regulated pathway in the secretory protein chromogranin A. J Cell Sci.

[14] Crippa L, Bianco M, Colombo B, Gasparri AM, Ferrero E, Loh YP, Curnis F, Corti A (2013). A new chromogranin A-dependent angiogenic switch activated by thrombin. Blood.

[15] Helle KB, Corti A (2014). Chromogranin A: a paradoxical player in angiogenesis and vascular biology. Cell Mol Life Sci.

[16] Maestroni S, Maestroni A, Ceglia S, Tremolada G, Mancino M, Sacchi A, Lattanzio R, Zucchiatti I, Corti A, Bandello F, Zerbini G (2015). Effect of chromogranin A-derived vasostatin-1 on laser-induced choroidal neovascularization in the mouse. Acta Ophthalmol.

[17] Theurl M, Schgoer W, Albrecht K, Jeschke J, Egger M, Beer AG, Vasiljevic D, Rong S, Wolf AM, Bahlmann FH, Patsch JR, Wolf D, Schratzberger P, Mahata SK, Kirchmair R (2010). The neuropeptide catestatin acts as a novel angiogenic cytokine via a basic fibroblast growth factor-dependent mechanism. Circ Res.

[18] Bianco M, Gasparri AM, Colombo B, Curnis F, Girlanda S, Ponzoni M, Bertilaccio MT, Calcinotto A, Sacchi A, Ferrero E, Ferrarini M, Chesi M, Bergsagel PL, Bellone M, Tonon G, Ciceri F (2016). Chromogranin A is preferentially cleaved into pro-angiogenic peptides in the bone marrow of multiple myeloma patients. Cancer Res.

[19] Ferrero E, Scabini S, Magni E, Foglieni C, Belloni D, Colombo B, Curnis F, Villa A, Ferrero ME, Corti A (2004). Chromogranin A protects vessels against tumor necrosis factor alpha-induced vascular leakage. FASEB J.

[20] Dondossola E, Crippa L, Colombo B, Ferrero E, Corti A (2012). Chromogranin A regulates tumor self-seeding and dissemination. Cancer Res.

[21] Chiorazzi N, Rai KR, Ferrarini M (2005). Chronic lymphocytic leukemia. N Engl J Med.

[22] Bichi R, Shinton SA, Martin ES, Koval A, Calin GA, Cesari R, Russo G, Hardy RR, Croce CM (2002). Human chronic lymphocytic leukemia modeled in mouse by targeted TCL1 expression. Proc Natl Acad Sci U S A.

[23] Bertilaccio MT, Scielzo C, Simonetti G, Ponzoni M, Apollonio B, Fazi C, Scarfo L, Rocchi M, Muzio M, Caligaris-Cappio F, Ghia P (2010). A novel Rag2−/−gammac−/−-xenograft model of human CLL. Blood.

[24] Corti A, Longhi R, Gasparri A, Chen F, Pelagi M, Siccardi AG (1996). Antigenic regions of human chromogranin A and their topographic relationships with structural/functional domains. Eur J Biochem.

[25] Ratti S, Curnis F, Longhi R, Colombo B, Gasparri A, Magni F, Manera E, Metz-Boutigue MH, Corti A (2000). Structure-activity relationships of chromogranin A in cell adhesion. Identification of an adhesion site for fibroblasts and smooth muscle cells. J Biol Chem.

[26] Schneider F, Bach C, Chung H, Crippa L, Lavaux T, Bollaert PE, Wolff M, Corti A, Launoy A, Delabranche X, Lavigne T, Meyer N, Garnero P, Metz-Boutigue MH (2012). Vasostatin-I, a chromogranin A-derived peptide, in non-selected critically ill patients: distribution, kinetics, and prognostic significance. Intensive Care Med.

[27] Belloni D, Scabini S, Foglieni C, Veschini L, Giazzon A, Colombo B, Fulgenzi A, Helle KB, Ferrero ME, Corti A, Ferrero E (2007). The vasostatin-I fragment of chromogranin A inhibits VEGF-induced endothelial cell proliferation and migration. FASEB J.

[28] Dondossola E, Gasparri AM, Colombo B, Sacchi A, Curnis F, Corti A (2011). Chromogranin A restricts drug penetration and limits the ability of NGR-TNF to enhance chemotherapeutic efficacy. Cancer Res.

[29] Pruneri G, Ponzoni M, Ferreri AJ, Decarli N, Tresoldi M, Raggi F, Baldessari C, Freschi M, Baldini L, Goldaniga M, Neri A, Carboni N, Bertolini F, Viale G (2002). Microvessel density, a surrogate marker of angiogenesis, is significantly related to survival in multiple myeloma patients. Br J Haematol.

