# Supplementary material for: Inhibition of chronic lymphocytic leukemia progression by full-length chromogranin A and its N-terminal fragment in mouse models
Source: Oncotarget. 2016 May 17;7(27):41725–36. doi: 10.18632/oncotarget.9407 (PMC5173091; doi:10.18632/oncotarget.9407)
Supplement: Supplementary file 1 [file oncotarget-07-41725-s001.pdf]

SUPPLEMENTARY DATA

Patients and plasma samples

Peripheral blood plasma samples were obtained from 37 patients with CLL at diagnosis (males/females, 23/14; stable/progressive disease, 28/9; age, 66.2 ± 12.2 years (mean ± SD)). Leukemic cells from these patients have been characterized for biological markers as CD38, ZAP70 and IGHV gene mutational status. Four patients (11.1%) were under treatment with proton pump inhibitors (PPI) and six patients (16.2%) had renal failure (RF), two factors that may enhance the levels of circulating CgA. Control plasma samples were obtained from 27 normal donors (males/females, 13/14; age, 58.6 ± 14.1 years (mean ± SD)) that were not taking proton pump inhibitors (Supplemental Table 1). All plasma samples were stored at -80 °C until analysis.

In vivo studies with Eμ-TCL1 transgenic mice

Sera from heterozygous Eμ-TCL1 transgenic mice (18), on a C57BL/6 background, or non-transgenic littermates were taken at different ages (two, six and ten months) and tested by CgA-ELISA. Eμ-TCL1 mice (3-month-old) were injected intra-peritoneum (i.p.) bi-weekly for 2 months with 1.5 μg/mouse of CgA in physiological solution containing 100 μg/ml of human serum albumin, or with vehicle alone. Leukemic cells in peripheral blood and bone marrow (BM), collected at sacrifice, were detected by FACS analysis. To this aim, cell suspensions obtained from blood and BM were incubated with Mouse BD Fc Block™ (BD Pharmingen) for 10 min at room temperature, to block Fc receptors, and stained with phycoerythrin-cyanine 7-labeled rat anti-mouse CD19 (clone 1D3) and allophycocyanin-labeled rat anti-mouse CD5 (clone 53-7.3) (BD Pharmingen). Leukemic cells were reported as percentage of $CD5^+$ $CD19^+$ cells/total $CD19^+$ cells.

All animals were treated in accordance with the European Union guidelines and with the approval of the Ethical Committee of the San Raffaele Scientific Institute.

In vivo studies with Rag2$^{-/-}\gamma c^{-/-}$ mice xenografted with MEC1 cells

Rag2$^{-/-}\gamma c^{-/-}$ female mice, on a BALB/c background (8-week-old), were challenged intravenously with 10^7 MEC1 cells, stably transfected with the green fluorescence protein, in 0.1 ml of physiological solution. Mice were treated daily i.v. with equimolar doses of CgA (0.3 μg), VS-1 (0.06 μg) or CgA_{1-373} (0.24 μg) in saline containing 100 μg/ml of human serum albumin, or with vehicle alone. Other mice were treated with a 30-fold higher dose of CgA (10 μg) in the same diluent. Mice were sacrificed at day 15, 16, 18 or 21, and dissected. The presence of MEC1 cells in cell suspensions obtained from kidneys, lungs, spleen and BM by mechanical tissue disaggregation, were analyzed by FACS using an anti-human CD19 antibody (clone J3-119, Beckman Coulter), taking advantage from the fact that this antibody does not cross react with murine CD19. To this aim, cells were incubated with Mouse BD Fc Block™ (BD Pharmingen) for 10 min at room temperature, stained with phycoerythrin-cyanine 7-labelled anti-human CD19 antibody (Beckman Coulter), and analyzed by FACS. In one experiment the kidneys of 3 mice per group were fixed in 4% paraformaldehyde for 1 h, left to incubate in three different sucrose solutions (15% for 2 h, 20% for 12 h, 30% for 12 h, at 4° C) and included in OCT. Tissue sections were then fixed with 4% paraformaldehyde, permeabilized with 0.1% Triton-X100, and stained with primary anti-GFP antibody (1:200 dilution, Molecular Probes) followed by secondary anti-rabbit antibody-Alexa Fluor 488 conjugate (1:500 dilution; Life Technologies). Nuclei were stained with DAPI (0.1 μg/ml). Cells were analysed using the Axioplan2 microscope (Zeiss, equipped with 20 × objective lenses) and images were captured with a digital camera and AxioVision Software.

Cell proliferation and apoptosis assays

MEC1 cell proliferation assay was performed as follows: MEC1 cells were stained with carboxyfluorescein diacetate succinimidyl ester (CFSE) (5 μM; Life Technologies) for 10 min at 37° C, washed, seeded into 96-well microtiter plates (2.5×10^4 cells/well), treated with various amounts of CgA in complete medium, and analyzed at different time points by FACS. CFSE dilution is an index of proliferation of MEC1 cells. The apoptosis assay was performed by measuring the percentage of Annexin-V (BD Pharmingen) and 7-amino-actinomycin D positive cells after incubation for 48 h with or without CgA in complete medium (0.5×10^6 cells/well, 12-well plates).

Cell migration assay

Cell migration assays were carried out as follows: MEC1 cells (0.5×10^6 cells in 100 μl of RPMI containing 0.1% of bovine serum albumin and different amounts of CgA) were seeded in the upper chamber of a transwell system (6.5 mm inserts, 5.0 μm pore-size filters, Corning Inc.). The lower chamber was filled with 600 μl of RPMI containing 0.1% of bovine serum albumin with or without 100 ng/ml of SDF-1α (PeproTech). Cells were left to migrate for 4 h at 37° C, 5% CO_2. Migrated cells, present

in the lower chamber, were counted by FACS analysis. To this aim, the entire medium in the lower chamber was collected, mixed with 10 µl of 5 µm sulfate latex beads (Invitrogen) diluted in PBS (1:150) and analyzed by FACS. A minimum of 10000 beads was acquired for each analysis. Each sample was analyzed in quadruplicate.

Trans-endothelial migration assay

Tumor cell trans-endothelial migration assay was performed using transwell systems (6.5 mm insert, 5.0 µm pore-size filters). HUVEC cells in EGM2 medium were seeded in the upper chamber of the transwell (0.5×10^5 cells/well) and incubated for 48 h at 37°C, 5% CO_2. Each insert was then washed with DPBS containing calcium chloride and magnesium chloride (Euroclone), and filled with MEC1 cell suspension (5×10^5 cells/100 µl of EBM2 containing 0.1% of bovine serum albumin and various amounts of CgA). The lower chamber was filled with EBM2 basal medium. The cells were left to migrate through the endothelial cell monolayer for 4 h. Cells migrated to the lower chamber were counted as described above for cell migration assays.

In vitro vascular permeability imaging assay

The effect of CgA on endothelial permeability was assessed using the "*In vitro vascular permeability imaging assay*" kit (EDM Millipore). HUVEC cells were seeded onto coverslips coated with biotinylated gelatin (0.5×10^5 cells/well) and incubated for 48 h at 37°C, 5% CO_2, until confluent monolayers were formed. The monolayers were then washed with DPBS and further incubated for 1 h in EBM2 basal medium containing 0.1% of human serum albumin and various amounts of CgA. Then TNFα was added to the supernatant and left to incubate for 4 h. Each sample was then fixed with 4% paraformaldehyde, stained with FITC-avidin according to manufacturer's instructions, and analyzed using a fluorescence microscope. The binding of FITC-avidin was quantified by measuring the number of positive pixel in each image taken with 20× objective after thresholding the FITC signal, using the imaging software ImageJ (National Institutes of Health, Bethesda, MD, USA). Three independent experiments were performed (2 slides/condition, 5 fields/slide for each experiment).

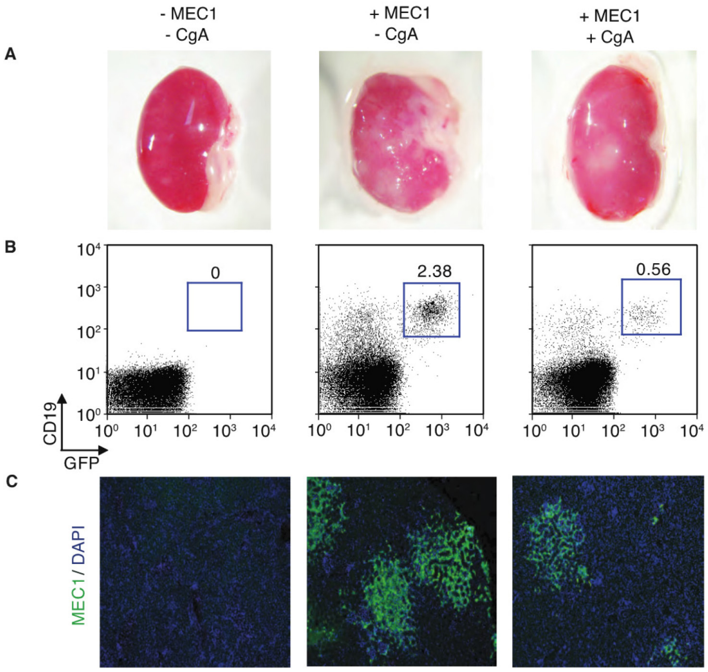

Supplemental Figure S1: Kidney infiltration by MEC1 cells expressing GFP in the MEC1 xenograft model of CLL. The experiment was performed as described for EXP-2 of Figure 3. Mice were sacrificed and dissected at day 16. The kidneys were then dissected, photographed and analyzed by FACS and immunofluorescence microscopy. **A.** Representative photographs of kidneys from the indicated experimental groups. **B.** FACS analysis of cell suspension obtained from kidneys after mechanical disaggregation with a cell strainer. Cells were stained with phycoerytrin-cyanine 7-labelled anti-human CD19 antibody (Beckman Coulter) and analyzed by FACS. The percentages of GFP/CD19 double-positive cells as a consequence of the different treatments are indicated (insets). **C.** Immunofluorescence microscopy of kidney tissue sections stained with anti-GFP antibodies to detect infiltrated MEC1-GFP cells (green) (see *Supplemental Methods*). Cell nuclei were counter-stained with DAPI (blu).

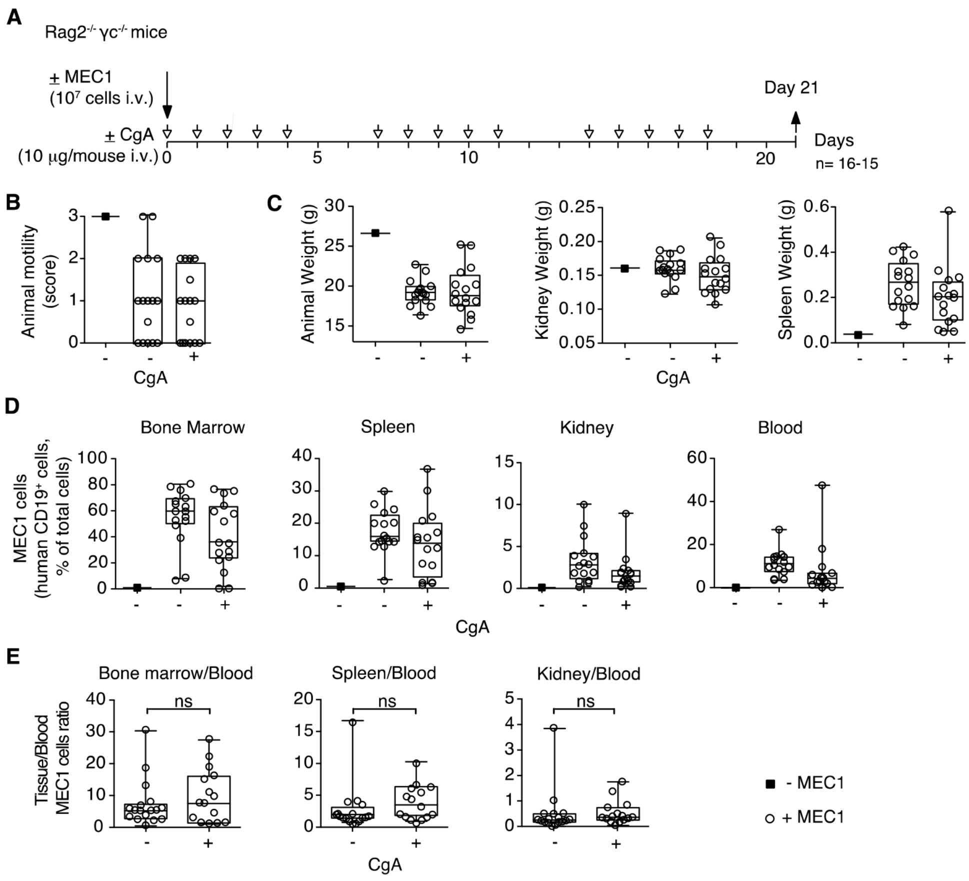

Supplemental Figure S2: Effect of high-dose CgA on organ infiltration by leukemic cells and disease progression in the MEC1 model. **A.** Schematic representation of the experiment. Rag2$^{-/-}$γc$^{-/-}$ mice, injected without (■) or with (○) MEC1 cells (i.v.), were treated with 10 µg of CgA (+) or with vehicle (-) at the indicated time, and sacrificed at day 21. **B.** Animal motility at day 21 before killing (see the legend of Figure 3 for score explanation). **C.** Animal weight, kidney and spleen weights at day 21. **D.** Quantification of MEC1 cells in bone marrow, spleen, kidney and blood, by FACS analysis (see legend of Figure 3). **E.** The tissue/blood ratios of MEC1 cells in different organs are shown. (B-E) Box-plots with median, interquartile and 5-95 percentile values. Differences between groups treated and untreated with CgA were non significant by *t* test, two-tailed.

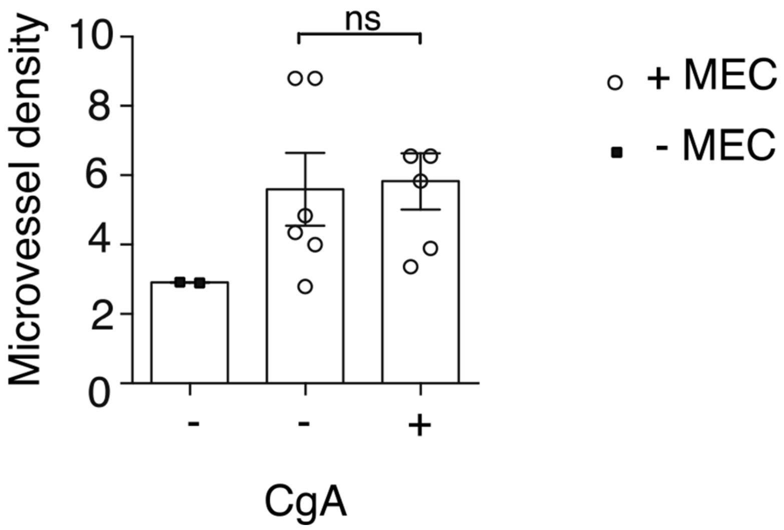

Supplemental Figure S3: Immunohistochemical analysis of BM microvascular density. Rag2$^{-/-}\gamma c^{-/-}$ mice, injected without or with MEC1 cells (i.v.), were treated with vehicle or with 0.3 µg of CgA$_{1\text{-}439}$ and sacrificed at day 21. We used the anti-mouse CD34 (Biolegend, clone MEC14.7) monoclonal antibody to calculate BM microvessel density by immunohistochemistry on whole sections, as previously described [29].

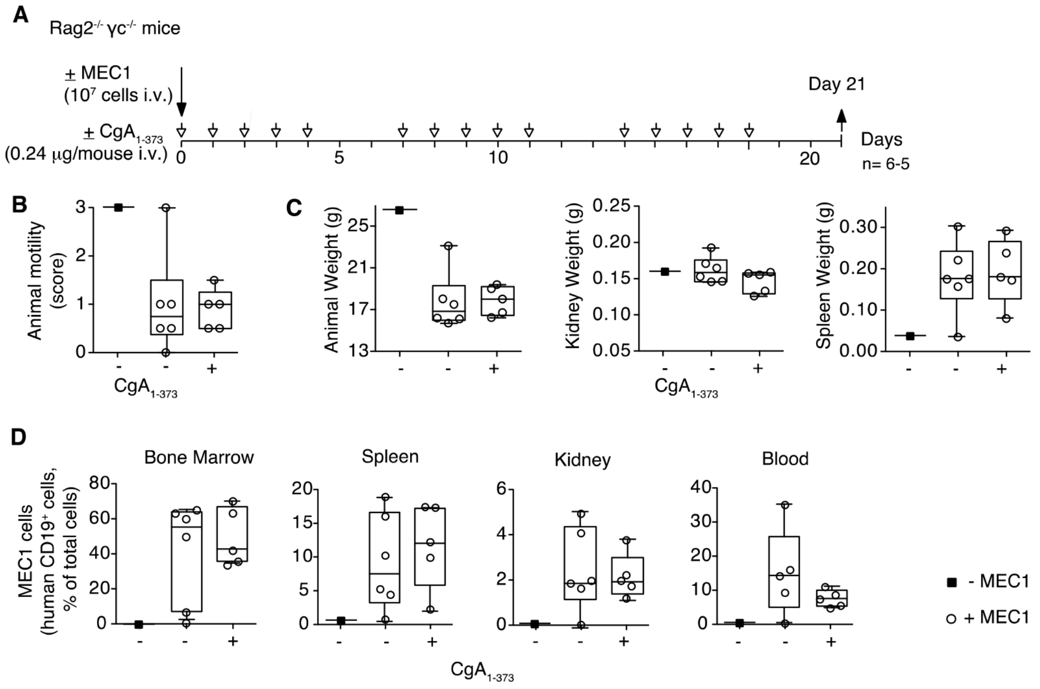

Supplemental Figure S4: Effect of low-dose CgA_{1-373} on organ infiltration by leukemic cells and disease progression in the MEC1 model. A. Schematic representation of the experiment. $Rag2^{-/-}\gamma c^{-/-}$ mice, injected without (■) or with (○) MEC1 cells (i.v.), were treated with vehicle (-) or with 0.24 µg of CgA_{1-373} (+) at the indicated time, and sacrificed at day 21. **B.** Animal motility at day 21 before killing (see the legend of Figure 3 for score explanation). **C.** Animal weight, kidney and spleen weights at day 21. **D.** Quantification of MEC1 cells in bone marrow, spleen, kidney and blood, by FACS analysis (see legend of Figure 3). (B-D) Box-plots with median, interquartile and 5-95 percentile values. Differences between groups treated and untreated with CgA were non significant by *t* test (two-tailed).

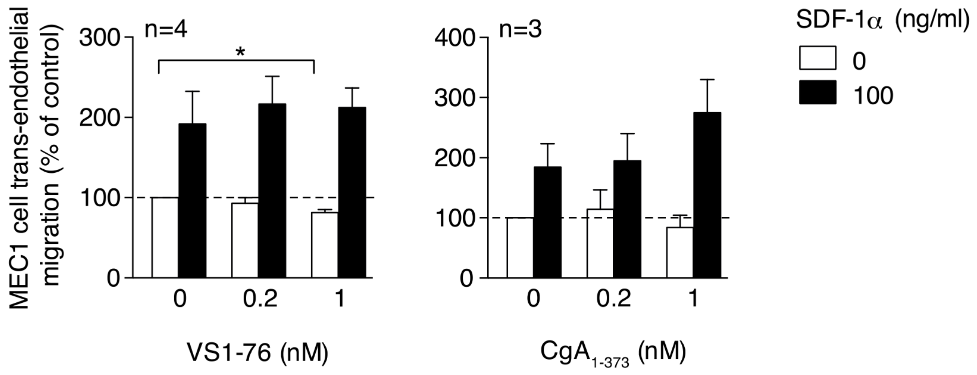

Supplemental Figure S5: Effect of VS-1 and CgA_{1-373} on MEC1 cell trans-endothelial migration. Effect of VS-1 and CgA_{1-373} on migration of MEC1 cells through endothelial monolayers cultured in trans-well systems. The upper and lower chambers of trans-well systems, pre-coated with HUVECs, were filled with different amounts of VS-1 (left panel) or CgA_{1-373} (right panel) and 0 or 100 ng/ml SDF-1α, respectively. Cells migrated to the lower side of the filter after 4 h of incubation, were counted by FACS (see *Supplemental Methods*). Bars, mean ± SEM of 3-4 independent experiments as indicated (n), each in quadruplicate (*, $P < 0.05$ by two tailed *t* test).

Supplementary Table S1: Demographic and clinical data regarding the study population (CLL patients and normal subjetcs)

Subject	n	(%)
**Patients**		
Total	37	
Male/Female	23/14	
Age: 66 years (median); 60-74 (25th-75th percentile)		
• Age <60 years	8	(21.6)
• Age 60-70 years	16	(43.2)
• Age >70 years	13	(35.1)
**Rai stage at diagnosis**		
• 0	19	(65.5)
• I	8	(27.6)
• II	2	(6.9)
• III-IV	0	(0)
**Clinical course**		
• Stable	28	(75.7)
• Progressive	9	(24.3)
**CD38 expression**		
• Positive (≥30%)	6	(19.4)
• Negative (<30%)	25	(80.6)
• Missing	6	-
**IGHV[(a)] mutational status**		
• Mutated	18	(62.1)
• Unmutated	11	(37.9)
• Not evaluable	8	-
**ZAP70 expression,**		
• Positive (≥20%)	11	(57.9)
• Negative (<20%)	8	(42.1)
• Missing	18	-
**Genomic aberrations**		
• Del13q	16	(55.2)
• Normal	6	(20.7)
• Trisomy 12	3	(10.3)
• Del11q	4	(13.8)
• Del17p	0	(0)
• Missing	8	-
**Normal subjects**		
Total	27	
Male/Female	13/14	
Age: 62 years (median); 51-71(25th-75th percentile)		

[(a)]Immunoglobulin heavy chain variable region genes.

Supplementary Table S2: Multi-analyte profiling of the supernatants of MEC1 cells treated with or without CgA (5 nM)[(a)]

See Supplementary File 1
