# Supplementary material for: Inhibition of chronic lymphocytic leukemia progression by full-length chromogranin A and its N-terminal fragment in mouse models
Source: Oncotarget. 2016 May 17;7(27):41725–36. doi: 10.18632/oncotarget.9407 (PMC5173091; doi:10.18632/oncotarget.9407)
Supplement: Supplementary file 2 [file oncotarget-07-41725-s002.docx]

| **Supplemental Table 2. Multi-analyte profiling of the supernatants of MEC1 cells treated with or without CgA (5 nM)^(a)^** | | | | | | | | | | | | | |
| --- | --- | --- | --- | --- | --- | --- | --- | --- | --- | --- | --- | --- | --- |
| **-CgA** | | | | | | | | | **+ CgA** | | | | |
| **Analyte** | **units** | | **D.L.^(b)^** | | **(mean + S.D.)** | | **(mean + S.D.)** | | | **∆ (%)** | | **p value** | |
| Alpha-2-Macroglobulin (A2Macro) | | µg /mL | | 0.1 | | < D.L. | | < D.L. | | |  | |  |
| Alpha-1-Antitrypsin (AAT) | | ng/mL | | 0.015 | | 0.08 + 0.02 | | 0.14 + 0.09 | | | 67.0 | | ns |
| Beta-2-Microglobulin (B2M) | | µg/mL | | 0.0003 | | 0.17 + 0.01 | | 0.20 + 0.01 | | | 11.8 | | ns |
| Brain-Derived Neurotrophic Factor (BDNF) | | ng/mL | | 0.004 | | < D.L. | | < D.L. | | |  | |  |
| Complement C3 (C3) | | ng/mL | | 0.018 | | 0.82 + 0.04 | | 0.82 + 0.07 | | | 0.1 | | ns |
| C-Reactive Protein (CRP) | | ng/mL | | 0.0001 | | < D.L. | | < D.L. | | |  | |  |
| Eotaxin-1 | | pg/mL | | 20 | | < D.L. | | < D.L. | | |  | |  |
| Factor VII | | ng/mL | | 0.56 | | < D.L. | | < D.L. | | |  | |  |
| Fibrinogen | | ng/mL | | 0.088 | | < D.L. | | < D.L. | | |  | |  |
| Ferritin (FRTN) | | ng/mL | | 0.013 | | 3.73 + 0.11 | | 3.62 + 0.40 | | | -2.9 | | ns |
| Granulocyte-Macroph. Colony-Stimul. Factor (GM-CSF) pg/mL | | | | 1.786 | | 4.17 + 0.46 | | 7.11 + 0.22 | | | 70.2 | | < 0.001 |
| Haptoglobin | | ng/mL | | 0.035 | | < D.L. | | < D.L. | | |  | |  |
| Intercellular Adhesion Molecule 1 (ICAM-1) | | ng/mL | | 0.478 | | 2.88 + 0.23 | | 2.71 + 0.33 | | | -5.9 | | ns |
| Interferon gamma (IFNγ) | | pg/mL | | 0.376 | | 0.98 + 0.20 | | 1.21 + 0.12 | | | 23.3 | | ns |
| Interleukin-1 alpha (IL-1α) | | ng/mL | | 0.002 | | 0.002 + 0.001 | | 0.003 + 0.001 | | | 64.2 | | ns |
| Interleukin-1 beta (IL-1β) | | pg/mL | | 0.358 | | < D.L. | | < D.L. | | |  | |  |
| Interleukin-10 (IL-10)  Interleukin-12 Subunit p40 (IL-12p40) | | pg/mL ng/mL | | 0.278 0.022 | | 587.66 + 16.16  < D.L. | | 593 + 31.24  < D.L. | | | 0.9 | | ns |
| Interleukin-12 Subunit p70 (IL-12p70) | | pg/mL | | 4.620 | | < D.L. | | < D.L. | | |  | |  |
| Interleukin-15 (IL-15) | | ng/mL | | 0.088 | | < D.L. | | < D.L. | | |  | |  |
| Interleukin-17 (IL-17) | | pg/mL | | 1.316 | | < D.L. | | < D.L. | | |  | |  |
| Interleukin-18 (IL-18) | | pg/mL | | 2.58 | | 3.25 + 1.50 | | 3.94 + 0.95 | | | 21.1 | | ns |
| Interleukin-1 receptor antagonist (IL-1ra) | | pg/mL | | 25 | | < D.L. | | < D.L. | | |  | |  |
| Interleukin-2 (IL-2) | | pg/mL | | 1.58 | | 2.31 + 0.62 | | 1.87 + 0.98 | | | -19.0 | | ns |
| Interleukin-23 (IL-23) | | ng/mL | | 0.164 | | < D.L. | | < D.L. | | |  | |  |
| Interleukin-3 (IL-3) | | ng/mL | | 0.004 | | 0.004 + 0.001 | | 0.01 + 0.001 | | | 34.5 | | ns |
| Interleukin-4 (IL-4) | | pg/mL | | 3.6 | | 19.4 + 1.42 | | 18.57 + 3.21 | | | -4.3 | | ns |
| Interleukin-5 (IL-5) | | pg/mL | | 0.694 | | < D.L. | | < D.L. | | |  | |  |
| Interleukin-6 (IL-6) | | pg/mL | | 0.823 | | 41.23 + 1.30 | | 82.23 + 7.90 | | | 99.4 | | < 0.001 |
| Interleukin-7 (IL-7) | | pg/mL | | 1.528 | | 5.05 + 0.94 | | 5.72 + 0.40 | | | 13.3 | | ns |
| Interleukin-8 (IL-8) | | pg/mL | | 0.376 | | 2116.66 + 603.68 | | 3373.33 + 410.04 | | | 59.4 | | < 0.05 |
| Monocyte Chemotactic Protein 1 (MCP-1) | | pg/mL | | 0.956 | | 1370 + 345.98 | | 1900 + 315.12 | | | 38.7 | | ns |
| Macrophage Inflammatory Protein-1 alpha (MIP-1α) | | pg/mL | | 4.58 | | 3090 + 50 | | 3146.67 + 45.09 | | | 1.8 | | ns |
| Macrophage Inflammatory Protein-1 beta (MIP-1β) | | pg/mL | | 2.44 | | 5106.66 + 282.9 | | 5736.67 + 255.8 | | | 12.3 | | < 0.05 |
| Matrix Metalloproteinase-2 (MMP-2) | | ng/mL | | 1.2 | | 15.4 + 4.71 | | 20.87 + 0.42 | | | 35.5 | | ns |
| Matrix Metalloproteinase-3 (MMP-3) | | ng/mL | | 0.01 | | < D.L. | | < D.L. | | |  | |  |
| Matrix Metalloproteinase-9 (MMP-9) | | ng/mL | | 4.14 | | < D.L. | | < D.L. | | |  | |  |
| T-Cell-Specific Protein RANTES (RANTES) | | ng/mL | | 0.0003 | | 0.79 + 0.03 | | 0.82 + 0.06 | | | 4.2 | | ns |
| Stem Cell Factor (SCF) | | pg/mL | | 19.56 | | < D.L. | | < D.L. | | |  | |  |
| Tissue Inhibitor of Metalloproteinases 1 (TIMP-1) | | ng/mL | | 0.019 | | 1.14 + 0.08 | | 1.26 + 0.06 | | | 11.1 | | ns |
| Tumor Necrosis Factor-α (TNFα) | | pg/mL | | 0.206 | | 96.17 + 3.80 | | 88.57 + 9.24 | | | -7.9 | | ns |
| Tumor Necrosis Factor-β (TNFβ) | | pg/mL | | 12.26 | | 1783.33 + 107.86 | | 1626.67 + 110.15 | | | -8.8 | | ns |
| Tumor Necrosis Factor Receptor 2 (TNFR2)  Vascular Cell Adhesion Molecule-1 (VCAM-1) | | ng/mL ng/mL | | 0.002 0.009 | | 1.48 + 0.074  1.58 + 0.046 | | 1.45 + 0.09  1.9 + 0.14 | | | -2.5 20.5 | | ns  < 0.05 |
| Vitamin D-Binding Protein (VDBP) | | ng/mL | | 0.028 | | < D.L. | | < D.L. | | |  | |  |
| Vascular Endothelial Growth Factor (VEGF) | | pg/mL | | 1.814 | | 22.5 + 2.88 | | 21.37 + 4.04 | | | -5.0 | | ns |
| von Willebrand Factor (vWF) | | µg/mL | | 0.001 | | 0.24 + 0.01 | | 0.24 + 0.028 | | | -1.1 | | ns |

^a)^ HUVEC cells (10^5^ cells/well) were seeded and incubated for 48 h at 37°C, 5% CO_2_. MEC1 cell suspension (5x10^5^ cells/well) was added to HUVEC monolayer and were treated with or without CgA (5 nM) in RPMI serum free medium for 24 h. Co-culture supernatants were analyzed by Human Inflammation MAP^TM^ 1.0 (Multi-Analyte ProfilingService, Rules Based Medicine) (n=3).

^b)^ D.L. Detection limit.
